# Supplementary material for: Screening and Identification of ssDNA Aptamers for Low-Density Lipoprotein (LDL) Receptor-Related Protein 6
Source: Molecules. 2023 Apr 30;28(9):3838. doi: 10.3390/molecules28093838 (PMC10180154; doi:10.3390/molecules28093838)
Supplement: Supplementary file 1 [file molecules-28-03838-s001.zip › Supplemental Information.pdf]

Table S1. Sequence information of the four mutant aptamers.

Name	Sequence (5′-3′)
MutLRP6-A1	AGCAGCACAGAGGTCAGATGCCGCAGGCAGTTTTCATTAGTCTCTATCCGTGACGGTATG CCTATGCGTTTTTCCGTGAA
MutLRP6-A2	AGCAGCACTTAGGTCAGATGGCCACATTAGTCTCACCACTACCTTTTTTCCTACCGCCGCC CTATGCGTGCTACCGTGAA
MutLRP6-A3	AGCAGCACAGAGGTTATTTGGCAGCTAAGCAGGCGGCTCACAAAACCATTCGCATGCGG CCCTATGCGTGCTTTTGTGAA
MutLRP6-A4	AGCAGCACAGAGGTCAGATGCGTTTTGCCTATTTTTATGACACAATCTTTTGGAGCGTAAC CTATGCGTGCTACCGTGAA

Note: The underlined parts are mutant nucleotides.

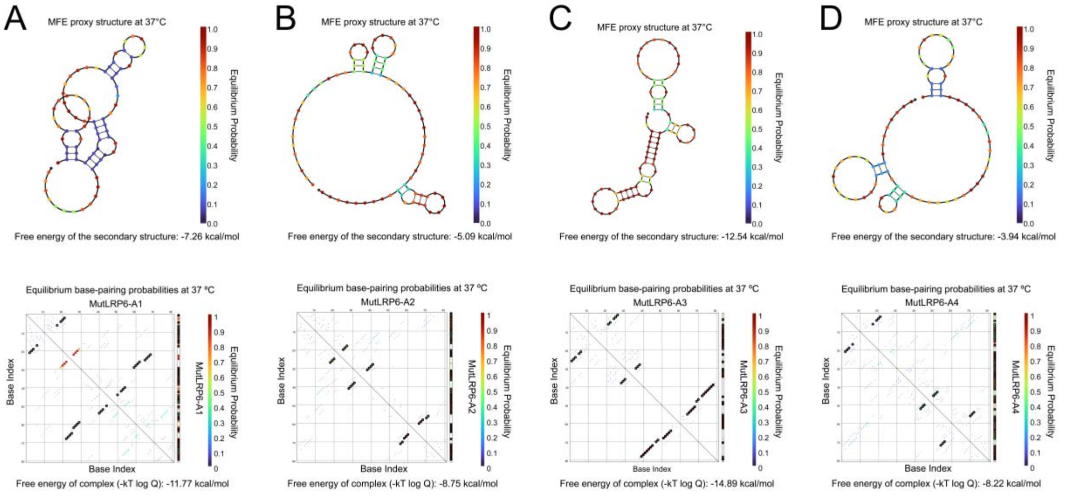

Figure S1. Secondary structure prediction of mutant candidate aptamers. **(A–D)** The secondary structures of MutLRP6-A1~MutLRP6-A4 were predicted by the NUPACK software.

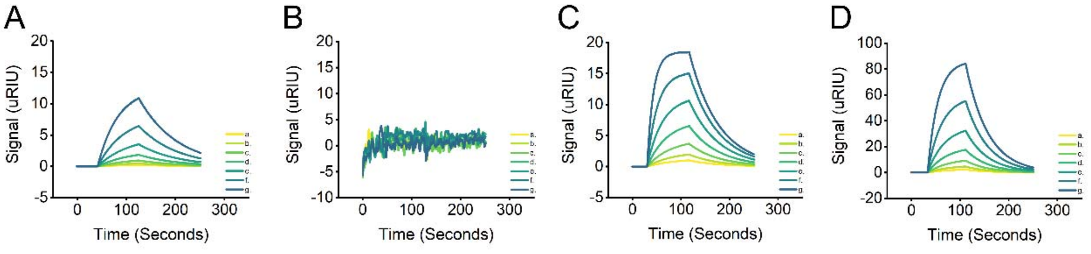

Figure S2. SPR characterizes mutant candidate aptamers' affinity and specificity. **(A–D)** respectively showed the SPR response spectrums of the binding of MutLRP6-A1~MutLRP6-A4 to LRP6.

Table S2. The binding kinetic parameters of mutant candidate aptamers and LPR6 were determined via SPR.

Name	Bmax ([Signal (uRIU)])	ka (1/ (M · s))	kd (1/s)	K_D (mol/L)	U-value: ka/kd (%)
MutLRP6-A1	26.8	1.13×10^{4}	1.29×10^{2}	1.13×10^{6}	9.9
MutLRP6-A2	-	-	-	-	-
MutLRP6-A3	23.43	6.21×10^{4}	1.66×10^{2}	2.68×10^{7}	6.2
MutLRP6-A4	159.22	2.60×10^{4}	2.20×10^{2}	8.47×10^{7}	3.4

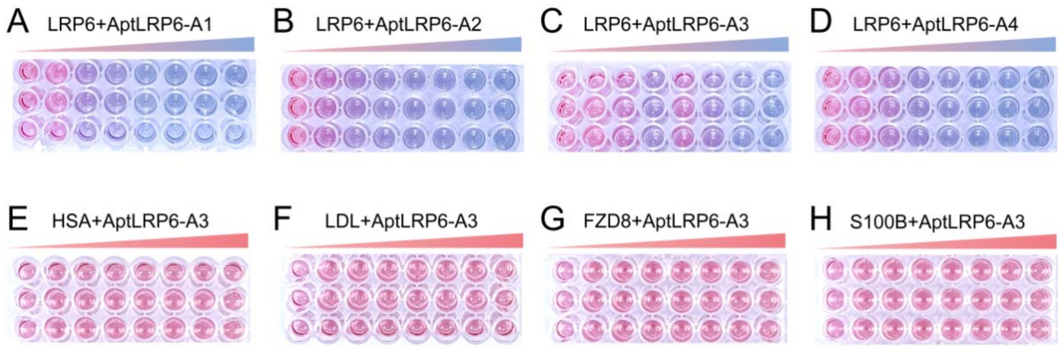

Figure S3. The affinities of candidate aptamers were verified using the gold nanoparticles (AuNPs) colorimetric method. **(A–D)** Color change of AuNPs solution after co-incubation of candidate aptamers AptLRP6-A1~AptLRP6-A4 with LRP6 protein, respectively. With the increase in the candidate aptamer concentration, the color of the AuNPs solution gradually changed from burgundy to blue. **(E–H)** Color change of AuNPs solution after co-incubation of candidate aptamer AptLRP6-A3 with control proteins HSA, LDL, FZD8, and S100B, respectively.

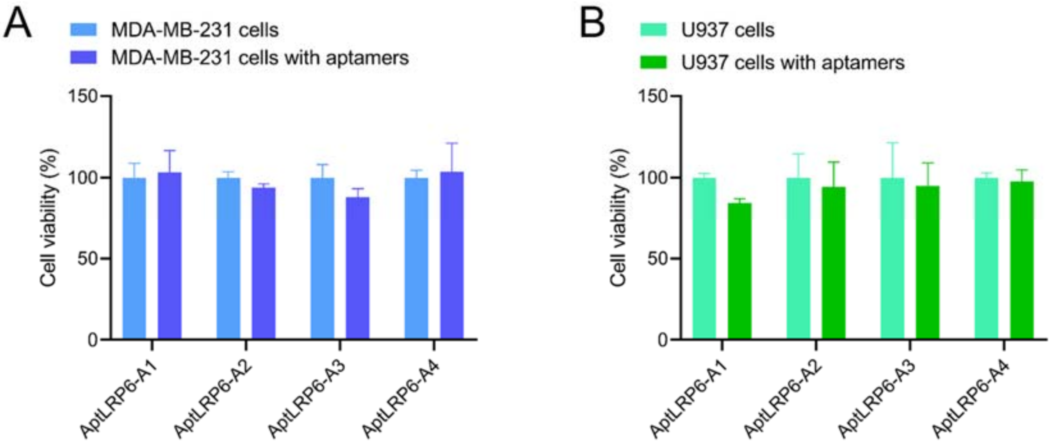

Figure S4. Effect of four candidate aptamers on cell growth. **(A)** Cell viability of 1 μmol/L candidate aptamers co-incubated with MDA-MB-231 cells for 24 h; **(B)** Cell viability of 1 μmol/L candidate aptamers co-incubated with U937 cells for 24 h.
